# Prospects of Gene Knockouts in the Functional Study of MAMP-Triggered Immunity: A Review

**DOI:** 10.3390/ijms21072540

**Published:** 2020-04-06

**Authors:** Benedict C. Offor, Ian A. Dubery, Lizelle A. Piater

**Affiliations:** Department of Biochemistry, University of Johannesburg, Auckland Park 2006, South Africa; benedictoffor@gmail.com (B.C.O.); idubery@uj.ac.za (I.A.D.)

**Keywords:** BAK1, innate immunity, KO, MAMPs, membrane raft, MTI, PRRs, T-DNA

## Abstract

Plants depend on both preformed and inducible defence responses to defend themselves against biotic stresses stemming from pathogen attacks. In this regard, plants perceive pathogenic threats from the environment through pattern recognition receptors (PRRs) that recognise microbe-associated molecular patterns (MAMPs), and so induce plant defence responses against invading pathogens. Close to thirty PRR proteins have been identified in plants, however, the molecular mechanisms underlying MAMP perception by these receptors/receptor complexes are not fully understood. As such, knockout (KO) of genes that code for PRRs and co-receptors/defence-associated proteins is a valuable tool to study plant immunity. The loss of gene activity often causes changes in the phenotype of the model plant, allowing in vivo studies of gene function and associated biological mechanisms. Here, we review the functions of selected PRRs, brassinosteroid insensitive 1 (BRI1) associated receptor kinase 1 (BAK1) and other associated defence proteins that have been identified in plants, and also outline KO lines generated by T-DNA insertional mutagenesis as well as the effect on MAMP perception—and triggered immunity (MTI). In addition, we further review the role of membrane raft domains in flg22-induced MTI in Arabidopsis, due to the vital role in the activation of several proteins that are part of the membrane raft domain theory in this regard.

## 1. Introduction

### 1.1. Plant Innate Immunity: An Overview

**Figure 1:**
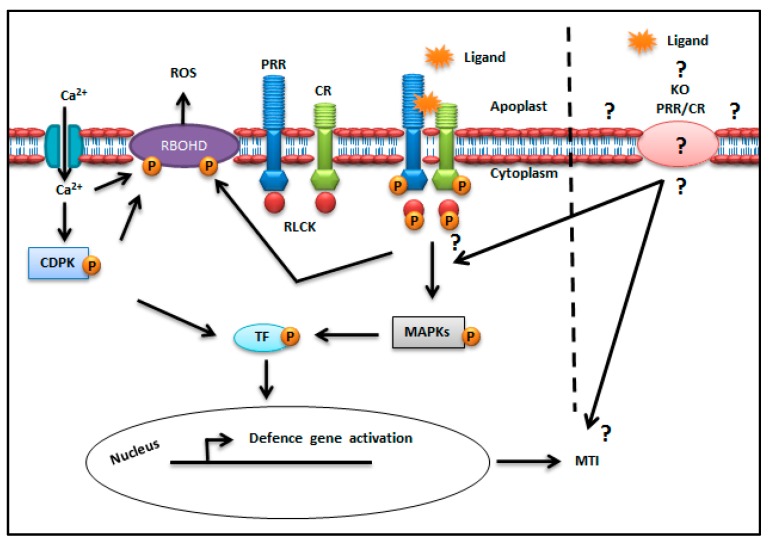
Generalised view of MAMP perception via PRRs, illustrating the potential impact of knockout (KO) effects that influence MAMP-triggered immunity. Upon MAMP (e.g., flg22) perception by a cognate PRR (e.g., in the case of FLS2 [28], the RLCK botrytis induced kinase1 (BIK1) dissociates from its complex with FLS2 and co-receptor BAK1. This leads to the activation of defence responses, such as ROS production, cytoplasmic Ca^2+^ influx, activation of downstream signalling kinases (MAPK and CDPK) and upregulation of defence genes. KO of the PRR and/or co-receptor (demarcated by the dotted line) may impair activation of downstream defence signalling, that could possibly lead to increased susceptibility of a plant to a pathogen. P = Phosphorylation, RBOHD = Respiratory burst oxidase homolog D, TF = Transcription factor, PRR = Pattern recognition receptor, CR = Co-receptor, MAMP = Microbe-associated molecular pattern, ROS = Reactive oxygen species, CDPK = Calcium-dependent protein kinase, MAPK = Mitogen-activated protein kinase, RLCK = Receptor-like cytoplasmic kinase, MTI = MAMP-triggered immunity.

Plants are continuously exposed to pathogen attack and this may lead to global losses in crop yields that, in turn, affect food security worldwide [1]. For instance, fungal-incited diseases are estimated to be responsible for 15–20% crop losses per annum [2]. Furthermore, oomycetal *Phytophtora infestans*, bacterial *Pseudomonas syringae* pv. *tomato* (*Pst*) DC3000 and fungal *Magnaporthe oryzae,* have been shown to cause diseases and losses of potato, tomato and rice, respectively [3,4,5]. Plants use wax layers, cutin and lignified cell walls, as well as preformed and inducible antimicrobial metabolites, to protect themselves against pathogen attack. Microbes that evade these barriers are confronted by an innate immune system, since plants lack adaptive immunity [6,7,8]. The primary defence response of plants is achieved through the perception of conserved signatures referred to as microbe-associated molecular patterns (MAMPs) by pattern recognition receptors (PRRs), localised in the plasma membrane, which leads to MAMP-triggered immunity (MTI) [8,9] (Figure 1). These plant PRRs mostly belong to the receptor-like kinase (RLKs) or receptor-like protein (RLPs) families. In this regard, RLKs contain an extracellular ligand-binding ectodomain, a transmembrane domain, and an intracellular serine/threonine (Ser/Thr) kinase domain [8,9,10]. The RLPs, on the other hand, have a similar architecture, but lack the intracellular protein kinase domain [9]. The cytoplasmic receptor-like kinases (RLCKs) are not able to perceive MAMPs, because they lack an extracellular domain, but can interact with RLKs and participate in phosphorylation cascades, thereby leading to the activation of downstream innate immune response [8,9,11].

MTI controls host microbial colonisation through the secretion of antimicrobial defence chemicals and nutrient deprivation of the attacking pathogen [12]. Several MAMPs such as epitopes from bacterial flagellin, elongation factor-thermo unstable (EF-Tu) and fungal chitin, have received much attention [13,14,15]. Other notable MAMPs under investigation are fungal ergosterol, ethylene-inducing xylanase (EIX), elicitin proteins, oomycetes β-glucan, bacterial cold shock protein (CSP), peptidoglycan (PGN), lipopolysaccharides (LPS), and necrosis and ethylene-inducing peptide 1-like proteins (NLPs) from multiple bacterial, fungal and oomycetal species [7,8,16,17,18,19,20,21,22,23,24,25]. The details, however, of the molecular mechanisms underlying MAMP perception by plant PRRs are elusive and appear to be different in each case, due to the unique physico-chemical properties of each ligand, specifically with regard to the binding kinetics and associated responses of the ligand: receptor interactions.

### 1.2. Cellular Events Associated with Plant Defence

The defence signalling events that occur when a PRR perceives its associated MAMP are illustrated in Figure 1, and include the induction of defence responses, such as reactive oxygen species (ROS) production and elevation of cytosolic calcium ([Ca^2+^] _cyt_). Here, Ca^2+^ binds to the first EF-hand motif in the N-terminal domain of respiratory burst oxidase homolog D (RbohD) and triggers ROS production [26]. Calcium-dependent protein kinase (CDPK), activated by the influx of calcium into the cytosol, thus contributes in the RbohD-dependent ROS production and induction of defence genes [27]. As an example, FLS2, as a well-studied PRR, forms a ligand (flg22)-induced complex with the co-receptor (BAK1) and recruits a RLCK (botrytis induced kinase 1, BIK1) [28]. BIK1 directly phosphorylates RbohD and contributes to the production of ROS and other downstream defence signalling [26]. Subsequent activation of signal transduction events linked to mitogen-activated protein kinases (MAPKs) leads to increases in the expression of defence-related genes, biosynthesis of pathogenesis-related (PR)-proteins, cell wall strengthening and callose deposition [24,29,30,31,32].

In order to abrogate MTI or the plant’s primary defence responses, true microbes secrete effectors that interact with host targets, inhibit PRR-mediated signalling and cause infection, termed effector-triggered susceptibility (ETS). Pathogenic effectors may subdue MTI through different means, which include binding to PRRs and the disrupting of PRR-MAMP complexes, targeting PRR kinase domains to inhibit kinase activity, and trans-phosphorylation and autophosphorylation [33,34,35]. Additionally, effectors may bind PRR signal amplifiers like BAK1, and target transcription factors and MAPK signalling pathways [33,34,35]. In response, resistant plants carry polymorphic nucleotide-binding site leucine-rich repeat (NBS-LRR) resistance (R) proteins that recognise these effector-mediated perturbations. This triggers a secondary, more intense defence response, termed effector-triggered immunity (ETI), which is often associated with the hypersensitive response (HR) that protects plants against such pathogens [29,31,36,37]. Collectively, these processes are described by the zig-zag model of plant innate immunity, and demonstrate the co-evolutionary relationship between pathogens and host plants [29].

Impairment or abolishment of MTI may result in enhanced pathogen proliferation and increased plant susceptibility to pathogens (Figure 1) [33]. As such, gene knockout (KO) by e.g., transfer DNA (T-DNA) studies, where functional genes are made inoperative, represents a rational approach towards the investigation of MAMP perception by potential PRRs, as well as the interactions with cognate receptors and/or probable co-receptors. As such, in this review, we focus mainly on MAMPs from bacterial, fungal and oomycete pathogens, of which the cognate PRRs have been identified, and subsequent KO lines by the T-DNA mutagenesis used to study plant immunity.

## 2. T-DNA Knockout and Other Mutagenesis Technologies

Mutant plants generated by disrupting genes that code for PRRs have been widely adopted in plant research. Since the main objective in this field is to improve crop production, mutant analysis to assay gene disruption effects is a useful tool [38]. T-DNA insertion into an intron/exon (should the intron insertion not be affected by splicing mechanisms) will disrupt gene expression, a phenomenon commonly referred to as “knockout” or null mutations [39]. The completion of the Arabidopsis genome sequence [40] has led to exponential interest in the use of KO methodologies as a reverse genetics approach to study plant metabolism [41]. T-DNA insertional mutagenesis has undisputed performance in plant research, despite rivalry from other gene KO systems such as RNA interference (RNAi) [42], zinc finger nucleases (ZFNs) [43], transcription activator-like effector nucleases (TALENs) [44,45] and clustered regularly interspaced short palindromic repeats (CRISPR)/CRISPR-associated protein 9 (Cas9) technology [46,47].

Insertional mutagenesis has the advantage of targeting individual genes within a closely related family, such that the function of these can be investigated [41]. Transposon mutagenesis is part of insertional mutagenesis where mobile transposons are removed from an original genomic location to another genomic position, by the aid of the transposon-encoded transposase enzyme [48]. That may disrupt the gene-coding region and concomitant loss-of-function phenotypes of that particular gene. A transposon mutagenesis screen has a number of advantages, which include high throughput, easy identification and provision of information into an organism’s genetic network [48]. Nevertheless, transposon insertion also has a number of draw backs. In this regard, intronic insertion of a transposon can be spliced out, leading to unsuccessful or unstable insertional mutagenesis. Furthermore, cryptic splice sites in the transposon’s sequence could lead in the generation of truncated transcripts [48].

T-DNA, on the other hand, is a short segment of DNA that is transferred from a bacterial tumour inducing (Ti) plasmid to the plant genome, when a plant is infected by *Agrobacterium tumefaciens* [41,49,50]. T-DNA insertional mutagenesis helps in the functional identification of genes that are responsible for an observed plant phenotype. Pertaining hitherto, Table 1 and Table 2 show selected plant PRRs, BAK1 and other associated defence proteins, the T-DNA KO genes and roles in plant MTI. T-DNA does not only disrupt the genes into which it is inserted, but also serves as a marker in the identification of the mutant [50]. The subsequent PCR-based screening of genomic DNA is a simple tool to select homozygous plants for the T-DNA insertional mutagens [38,39]. Here, insertions in the target genes are detected by the use of a combination of gene-specific and T-DNA-specific primers. As such, in this review, we focus on T-DNA insertional mutagenesis of well-studied PRRs, co-receptors and other associated proteins, and discuss the contribution in the study of MTI.

T-DNA is flanked by 25 bp right border (RB) and left border (LB) sequences, respectively, that enables the replacement of native DNA with DNA of interest to enhance genetic engineering [51]. Successful transfer of the T-DNA into the host plant’s nuclear genome requires virulence (*vir*) genes (*vir*A, *vir*B, *vir*C, *vir*D, *vir*E, *vir*F, and *vir*G) [51,52]. VirD1 and VirD2 endonucleases, which cut the bottom part of the T-DNA border, release the single stranded DNA (T-strand) and attach to the 5′ end [53]. The T-strand is escorted through the cytoplasm into the nucleus by a complex (T-complex) that includes VirD2, VirE2 and VirF [51]. The T-DNA is then integrated into the plant genome in various orientations (full-length, truncated or multiple T-DNAs) and requires plant proteins [53]. Subsequently, T-DNA insertion alters both the genomic and epigenomic landscape of Arabidopsis T-DNA insertion lines [54]. The advantage of using T-DNA insertion is that there is no transposition after integration within the genome, and it is chemically and physically stable through multiple generations [39]. Additionally, mutations that are homozygously lethal can be obtained and maintained in a population in the form of heterozygous plants [50]. There are, however, also a number of disadvantages associated with T-DNA insertional mutagenesis. These include multiple T-DNA insertions, different insertion sizes and chromosomal rearrangements [54,55]. In addition, T-DNA insertion has been reported to induce the decreased expression of adjacent genes in Arabidopsis [56]. Another major limitation of T-DNA mutagenesis, like all gene disruption approaches, is the possibility of no phenotypic alteration due to gene duplication or redundancy, especially in genes required for early embryogenesis or gametophytic development [49,50]. Notably, over 70% of Arabidopsis genes are present in more than one copy [40,41]. To overcome these limitations, the generation of double or multiple mutations in a group of related genes is important in observing phenotypic alteration [49]. In this regard, expression patterns using specialised vectors with modified insertional elements can be used to identify redundant genes [50].

## 3. Membrane Raft Domains in the flg22-Mediated Defence Response

The Sanger and Nicholson fluid-mosaic model [57] describes the plasma membrane (PM) as comprised of a lipid bilayer, with integral and peripheral proteins randomly distributed within the membrane. Thereafter, the membrane raft concept, first introduced by Simons and Ikonen [58], defined specific regions of the plasma membrane with high concentrations of specific lipids (sphingolipids, sterols) and proteins that are involved in both membrane trafficking and signalling. Membrane rafts are formed through lipid-lipid interactions, where sphingolipids on the outer leaflet of the membrane interact with sterols, such as either cholesterol in mammalian cells or phytosterols in plant cells [58,59,60]. These microdomains are dynamic and highly organised at the nanoscale (10-200 nm in diameter) level within various parts of the membrane, and can be stimulated into larger and more stable raft domains by lipid-lipid—as well as protein-lipid—and protein-protein interactions [59,61,62]. Membrane rafts contain a high content of saturated fatty acids within the phospholipids, as well as cholesterol, compared to the non-raft regions of the PM, thereby producing a liquid-ordered (Lo) phase [63,64]. This tight packing of the lipids into a less fluid Lo phase confers detergent-resistant properties to membrane rafts [61]. At low temperature (4 °C), the Lo phase structures of rafts can be recovered from membrane fractions as a consequence of the tight packing of the lipids and insolubility to non-ionic detergent (Triton X100), and are therefore referred to as ‘detergent-insoluble membranes’ (DIMs) [61,65]. Functionally, membrane rafts play regulatory roles in signal transduction, endocytosis and exocytosis, cell adhesion, actin cytoskeleton organisation and cell trafficking [59,64,65,66].

Proteins associated with membrane rafts include G-proteins, protein kinases and glycophosphatidyl inositol (GPI)-anchored proteins that are involved in signal transduction [62,64,67]. As such, membrane rafts are believed to play vital roles as signal transduction platforms during plant biotic/abiotic stress [65], and are proposed to be the hub for PM-localised receptor proteins (like PRRs) and co-receptor complexes that are involved in MAMP perception and defence signalling in plants [65,66]. Consistently, the induction of proteins was observed in DIMs prepared from Arabidopsis, tobacco and rice, upon treatment with bacterial flg22, oomycetal cryptogein and fungal chitin, respectively [68,69,70]. Using quantitative proteomics, Kierszniowska et al. [67] identified proteins that are involved in signalling, such as receptor kinases, G-proteins and calcium signalling proteins in *Arabidopsis thaliana* rafts. Similarly, flg22 induced proteins, such as proton pump ATPases and RLKs including FLS2 in DIMs of *A. thaliana* cells [70].

**Figure 2:**
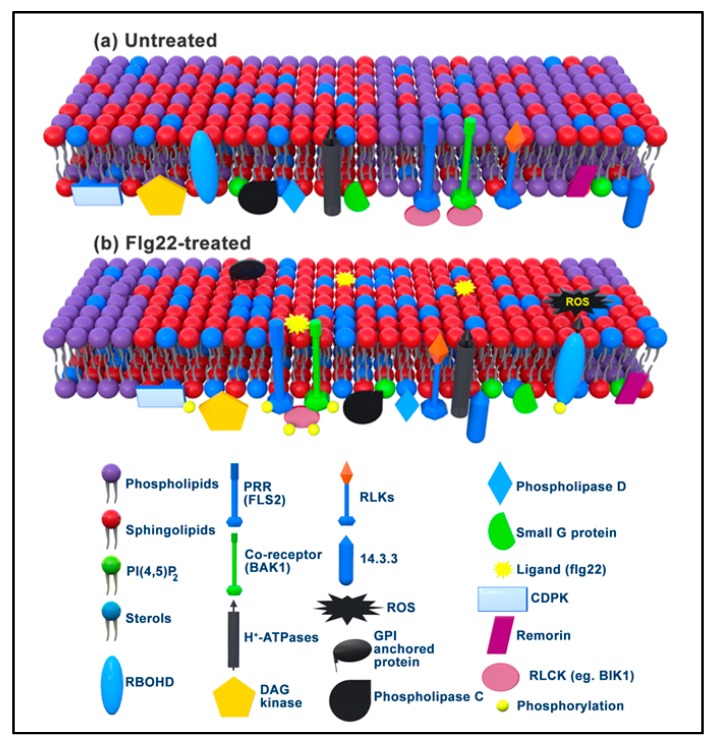
A model for localisation of PRRs in membrane rafts upon flg22 MAMP elicitation in *A. thaliana*. (**a**) Non-stimulated cells contain FLS2, BAK1 and other RLKs in non-raft domains (dominated by phospholipids), with inactive RbohD, PIP2-PLC, DGK typical raft protein markers (like remorin). (**b**) In flg22 treated cells, FLS2 forms a complex with BAK1 in DIMs (dominated by sphingolipids and sterols), that follows with recruitment of the RLCK BIK1 to the FLS2-BAK1 complex. Transphosphorylation events lead to RbohD activation and downstream defence signalling. Other components of DIM include CDPK, Phospholipase D (PLD), Phospholipase C (PLC), small G protein (Rac) and channel proteins (adapted from [28,65,66,73]).

Figure 2 demonstrates a model of the activation of the RbohD enzyme upon flg22 perception by FLS2 and subsequent signalling in raft domains of *A. thaliana* [64,65,66]. In non-treated *A. thaliana* cells, FLS2 is located in non-raft domains or detergent-soluble domains (DSMs), whereas inactive RbohD enzymes, phosphatidylinositol (4,5)-bisphosphate-specific phospholipase C (PIP2-PLC), diacylglycerol kinase (DGK) and typical raft proteins, such as remorin (REM), are localised inside rafts or DIMs. In pre-active states, both FLS2 and BAK1 are located in different regulatory protein complexes. For example, FLS2 is found in complex with the RLCK, BIK1, whereas BAK1 is associated in complex with BAK1-interacting receptor-like kinases (BIR1, BIR2 or BIR3) [28,71,72]. Immediately upon flg22 treatment of the cells, FLS2 shifts from DSMs to DIMs, forms a complex with BAK1, and recruits BIK1 that leads to the activation of RbohD enzymes through a series of phosphorylation events and concomitant downstream defence signalling. Thus, the recruitment of regulators of RbohD, such as the 14.3.3 protein, the Rac G protein (a small GTPase immunoregulator) and the phospholipid, phosphatidic acid (PA), to the N-terminus of RbohD, leads to its activation [28,65,66,68,69,70,73,74]. In addition, other proteins reported to be abundant in DIMs upon flg22 elicitation include channel proteins, RLKs, and transporters (e.g., ABC transporter and H^+^-ATPase AHA1) [73]. RLKs, including BAK1, have also been identified in the DIMs of tobacco suspension cells [75]. Although there has been much research interest in membrane rafts, many controversies exist regarding their existence and function [60], and more studies are needed to substantially understand their role in, and contribution to, plant defence signalling.

## 4. Pattern Recognition Receptors in MTI

**Figure 3:**
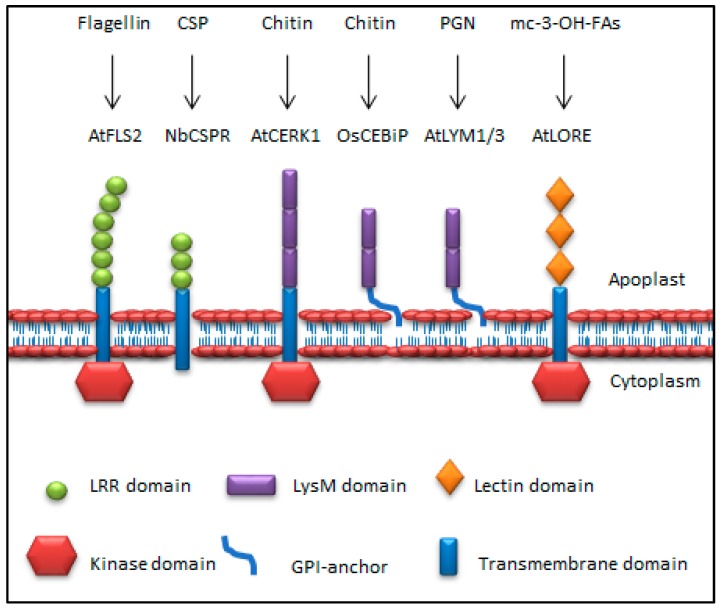
Structural configuration of plant PRRs. PRRs use different conserved external surfaces such as LRR-, LysM- or lectin domains to recognise MAMPs. Following, the transmembrane domain relays the perceived signal into the cell for the kinase domain to perform PRR activation, phosphorylation of downstream defence-related proteins and subsequent MTI induction. LRR = leucine-rich repeat, LysM = lysine motif, GPI = glycophosphatidyl inositol.

PRRs play key roles in plant defences against microbes. Figure 3 illustrates the different structural configurations of selected PRRs in plants, to demonstrate the different ectodomains that are involved in MTI. Several PRRs have been characterised in different plants, including EF-Tu receptor (EFR) and flagellin sensitive 2 (FLS2), which are both RLKs [14,76,77]. RLPs, such as the chitin elicitor-binding protein (CEBiP) identified in rice, require an additional protein (chitin elicitor receptor kinase 1; CERK1), with cytoplasmic kinase activity to trigger chitin defence signalling [78,79]. RLKs and RLPs are classified into subfamilies, depending on the motifs or domains of the ectodomains [36]. These domains include leucine-rich repeat (LRR), lysin M (LysM) or lectin (Lec), used to bind specifically to ligands [9,80]. A number of RLKs and RLPs use LRRs to bind MAMP epitopes. For example, FLS2, is a LRR-RLK that binds bacterial flg22 (an epitope in flagellin) and triggers immune defence signalling [76,81]. On the other hand, cold shock protein receptor (NbCSPR), a LRR-RLP, associates with BAK1 and, upon cold shock protein epitope (csp22) treatment, induces defence responses in *Nicotiana benthamiana* [82]. In tomato, cold shock receptor (CORE), a LRR-RLK, recognises bacterial csp22 and triggers defence responses [83]. These indicate the presence of two modes of CSP perception in the Solanaceae. Transgenic expression of NbCSPR in *A. thaliana* conferred csp22 responsiveness and resistance to *Pst* DC3000 bacteria. Other notable LRR-RLKs involved in MTI are rice XA21 and tomato FLS3, that recognise the bacterial peptides required for the activation of XA21-mediated immunity X (RaxX) and flgII-28, respectively [84,85]. Other examples of LRR-RLPs include Arabidopsis receptor-like protein 23 (RLP23) and tomato elicitin response (ELR), that recognise nlp20 and oomycetes elicitins [17,86].

PRRs with LysM ectodomains are also part of both RLK and RLP families. LysM-RLK CERK1 uses such motifs on its extracellular domain to interact with the MAMP chitin in *A. thaliana* [15], while LysM-containing RLPs, LYM1/LYM3, perceive PGN in *A. thaliana* [87]. Notably, LYM1/3 and LYM2 RLPs, which are Arabidopsis homologs of OsCEBiP, possess a unique glycophosphatidyl inositol (GPI) anchor used to attach the receptors in the plasma membrane bilayer [12,87,88]. Additional examples of LysM-RLKs include Arabidopsis LYK5 and LYK4, which are involved in fungal chitin perception and defence signalling [89,90], while members of LysM-RLPs include the rice LYP4 and LYP6, that recognise fungal chitin and bacterial PGN respectively [91].

The bulb-type (B-type) lectin S-domain (SD)-1 RLK, lipooligosaccharide-specific reduced elicitation (LORE), which is a lectin (Lec)-RLK, was reported to sense the bacterial *Xanthomonas* and *Pseudomonas* LPS, and to activate MTI in Arabidopsis [92]. However, recently Kutschera et al. [93] reported that 3-hydroxy fatty acids that co-purified with LPS induced a LORE-dependent defence response in *A. thaliana*. Thus, LORE is not the receptor for LPS. Relatedly, Sanabria et al. [94] proposed an LPS-responsive *N. tabacum* S-domain RLK (Nt-Sd-RLK) gene encoding conserved B-lectin, S- and PAN domains to be involved in LPS perception. These authors proposed that S-domain RLK MAMP perception and signal transduction could be via direct ligand recognition and binding of carbohydrate epitopes, or by indirect ligand-induced conformational changes, dimerisation or recruitment of a co-receptor to initiate phosphorylation events that lead to the activation of defence signalling [94,95,96,97].

Although this review is focused on mutant lines where specific PRRs have been knocked-out, it is important to note that most RLKs and RLPs do not function in isolation, but mostly act together with associated proteins that participate in defence signal transduction upon MAMP perception. In this regard, the RLK co-receptor BAK1 has been shown to be recruited upon MAMP perception to modulate defence signalling [96,98,99]. In addition to BAK1, other proteins might be recruited to the PRRs to form a functional recognition complex and regulate immune signalling [74,100]. For example, SOBIR1, an adaptor for LRR-RLPs, regulates cell death and innate immunity in Arabidopsis and tomato [101,102,103], while FERONIA (FER) and IMPAIRED OOMYCETE SUSCEPTIBILITY1 (IOS1), both malectin-like RLKs, form ligand-induced complexes with PRRs and regulate MTI in Arabidopsis [104,105].

Additional protein-protein interactions and motifs deserve some mention, particularly to highlight the need to regulate PRR activities, as well as turnover, in order to keep immune signalling in check. In this regard, an elicitor-responsive Armadillo repeat protein (GhARM) from cotton (*Gossypium hirsutum*) regulates cell wall-derived *Verticillium dahliae* elicitor responsiveness and contains three consecutive ARM repeats that, in association with certain RLKs or co-receptors, activate plant defence signalling [106]. Upon flg22 treatment, the Arabidopsis U-box E3 ubiquitin ligase, PUB13, uses a C-terminal ARM repeat domain to interact and ubiquitinate FLS2 in a BAK1-dependent manner [107]. Additionally, flg22-mediated FLS2 internalisation and endocytosis depends on cytoskeleton, receptor activation, and proteasome functions. Mutation at the threonine 867 of FLS2, proposed to bind flg22, showed impairment in flg22 signalling, as well as FLS2 endocytosis [108]. The Arabidopsis PUB13, a close ortholog of rice SPOTTED LEAF 11 (SPL11), is furthermore known to regulate cell death, defence and flowering time in a SA-dependent manner [109,110], as well as ubiquitinates LYSIN MOTIF RECEPTOR KINASE5 (LYK5) in vitro to regulate the turnover thereof [111]. The tomato (*Solanum lycopersicum*) homolog of PUB13, SIPUB13, on the other hand, works with group III members of ubiquitin-conjugating enzyme (E2s) to ubiquitinate FLS2 in vitro [112]. Negative regulation of MTI upon induction by several MAMPs has also been reported for the Arabidopsis E3 ubiquitin ligase triplets, PUB22, PUB23, and PUB24 respectively. Upon induction by MAMPs, including flg22 and chitin, *pub22/pub23/pub24* triple mutants displayed an increased ROS burst and strong upregulation of *OXI1* and *WRKY29* defence genes [113].

Conclusively, as an outcome of recent fundamental studies, results have shown that immune receptors can potentially be transferred or engineered to enhance MAMP and/or pathogen recognition, and quantitative responsiveness, to control plant diseases in crop [33,100,114,115].

## 5. Well-Studied MAMP Perception System(s)

### 5.1. Bacterial MAMPs

#### 5.1.1. Flagellin Perception

Flagellin is the main protein component of bacterial flagella, that acts as a MAMP in both plants and animals. Plants have a notably sensitive perception system for the highly conserved domain in the N-terminus of eubacterial flagellin [13]. The perception thereof, and particularly the N-terminus flagellin peptides, flg22 and flg15 from *Pseudomonas syringae* pv. *tabaci*, lead to the alkalinisation of the culture medium in suspension-cultured cells of *Lycopersicon peruvianum* (a wild relative of tomato), *A. thaliana*, potato and tobacco respectively [13]. Flagellin from the rice-incompatible strain, N1141 of *P. avenae*, was also shown to induce the hypersensitive cell death and accumulation of EL2 mRNA (elicitor-responsive gene) in cultured rice cells [116]. As mentioned, FLS2 is a LRR-RLK involved in the perception of the bacterial flagellin and immune responses in *A. thaliana* [76,96]. Furthermore, the recognition of bacterial flg22 by Arabidopsis FLS2 induces a FLS2-BAK1 complex formation and triggers defence signalling [96,98]. By using chemical cross-linking and immunoprecipitation techniques, Chinchilla et al. [117] showed that the specificity of flagellin perception and immune responses is mediated by the binding of FLS2 to flg22 in *A. thaliana*. The *fls2* mutants carrying T-DNA insertion in the flagellin receptor gene *FLS2* were more susceptible to *Pst* DC3000 compared to the wildtype (WT) [118]. Upon flg22 treatment, *FLS2* mutants (*fls2-101*), generated by the insertion of T-DNA at the third *FLS2* exon, showed impaired binding of the flg22 and reduced seedling growth inhibition [81].

As stated, flg22 induces a FLS2-BAK1 complex in a ligand-dependent manner, and leads to defence responses in *A. thaliana* [96]. This study showed that although *BAK1* mutants (*bak1-3* and *bak1-4* with T-DNA inserted in 5^th^ intron and 9^th^ exon, respectively) were defective in an oxidative burst generation, they did not affect the binding of flg22 to FLS2. Furthermore, flg22 induces recruitment of the U-box E3 ubiquitin ligases (PUB12 and PUB13), previously mentioned, to the FLS2-BAK1 complex and this causes FLS2 degradation and the concomitant attenuation of immune signalling in Arabidopsis [107]. Here, there was a decrease in FLS2 ubiquitination and *Pst* DC3000 infection in mutant plants (*pub12-2*, *pub13* and *pub12/13* double knockout), compared to WT controls. A recent study has furthermore shown that flagellin-sensing 3 (FLS3) directly and specifically binds flgII-28 (a second flagellin epitope distinct from flg22), which enhances immune responses in tomato [85]. Finally, it is also important to note that BAK1 is involved in the defence signalling activities of both FLS2 and FLS3 [85,96].

#### 5.1.2. EF-Tu Perception

Elongation factor-thermo unstable (EF-Tu), a prokaryotic elongation factor involved in the synthesis of proteins, is a bacterial MAMP that is recognised by the LRR-RLK, EFR, in Arabidopsis [14]. *N. benthamiana* normally lacks EF-Tu responsiveness, but achieved the ability to recognise the MAMP when EFR was transiently expressed in the plant [14]. Kunze et al. [119] showed that EFR binds directly to the acetylated N-terminus epitope of elf18 (the first amino acids of EF-Tu) and elicits innate immunity in Arabidopsis and other Brassicaceae species. The first acetylated 12 N-terminal amino acids (elf12) were, however, not able to elicit an immune response, but rather acted as a specific antagonist of elf18 [119]. Interestingly, even though EF-Tu induces MTI in rice, the elf18 peptide failed to trigger an immune response. However, another epitope, EFa50 (50-amino acid from central region of EF-Tu comprising Lys176 to Gly222) induced MTI responses, although the related PRR is still unknown [120]. Rice leaves treated with EFa50 induced early defence responses such as increased H_2_O_2_ and callose deposition, and triggered resistance to coinfection with pathogenic bacteria. In this regard, Arabidopsis *EFR* T-DNA insertion mutants (*efr-1* and *efr-2*), with both T-DNAs inserted in 1^st^ exon, were insensitive to EF-Tu and susceptible to the bacterium *Agrobacterium tumefaciens* [14] (Table 1). In fact, *efr* mutants did not show typical growth inhibition as for the WT, increased oxidative burst and ethylene biosynthesis or induced resistance to *Pst* DC3000 upon EF-Tu treatment. Jeworutzki et al. [121] reported involvement of EFR and FLS2 in the Ca^2+^-associated opening of plasma membrane anion channels during early bacterial flagellin and EF-Tu defence signalling in Arabidopsis mesophyll cells. Using electrophysiological approaches, they showed that the *efr-1* was defective in the membrane potential depolarisation, which is indispensable for cytosolic calcium influx in response to elf18 treatment.

#### 5.1.3. LPS Perception

LPS is an amphipathic molecule found on the outer membrane of Gram-negative bacteria and protects the organism against antimicrobial compounds found in the environment [23,122]. This MAMP is involved in the bacterial adhesion and induction of defence-related responses in both mammals and plants [123]. In the former, LPS recognition is orchestrated by lipopolysaccharide binding protein (LBP), before recruitment into a complex comprising soluble myeloid differentiation protein 2 (MD-2), membrane attached cluster of differentiation 14 (CD14), and the transmembrane Toll-like receptor 4 (TLR4), thereby leading to mammalian defence activation against LPS [6,124]. LBP and bactericidal/permeability-increasing protein (BPI) thus play a vital role in regulation of immune responses against LPS in mammals [124,125]. As such, the mechanism by which LPS is recognised has been well-studied in animals, however, in plants, the mechanism of perception and recognised moiety/epitope(s) of LPS is still debateable.

In this regard, LPS, as well as its lipid A moiety from *Burkholderia cepacia, Xanthomonas campestris and P. syringae*, trigger the upregulation of genes involved in immunity and defence [126,127,128], with lipid A speculated to be the major elicitor in *A. thaliana* [129]. LPS has furthermore been shown to trigger defence responses, such as the generation of nitrogen oxide (NO), ROS, elevation of cytoplasmic Ca^2+^ concentration, stomatal closure and the expression of pathogenesis-related (PR) genes in Arabidopsis, tobacco and rice suspension cells [92,130,131,132,133]. Ranf et al. [92] earlier reported that LPS from *Xanthomonas* and *Pseudomonas* are sensed by the bulb-type (B-type) lectin S-domain (SD)-1 RLK lipooligosaccharide-specific reduced elicitation (LORE) and that *LORE* mutants showed defects in the LPS-induced elevation of cytosolic calcium, ROS and defence gene (*AtFRK1*) expression. Additionally, Shang-Guan et al. [129] reported the existence of two ROS production phases, characterised by a weak initial and second stronger ROS generation in *A. thaliana*. T-DNA insertion *LORE* mutants, however, showed little or no difference in second phase ROS production compared with WT, suggesting another LPS receptor(s). In contradiction to the original LORE study, a recent report implicated bacterial 3-hydroxy fatty acids in LORE-dependent induction of immune response in Arabidopsis [93]. Here, LORE could not sense LPS that was repurified to remove free 3-hydroxy fatty acids, indicating that LORE is not the receptor for LPS.

Using *B. cepacia* LPS-affinity capture strategies, Vilakazi et al. [134] and Baloyi et al. [135] identified BAK1 and other defence response proteins associated with the plasma membrane fraction in LPS-treated *A. thaliana*. Additionally, Arabidopsis LBP/BPI related-1 (AtLBR-1) and LBP/BPI related-2 (AtLBR-2) were shown to bind to both rough and smooth LPS, and regulate the expression of the *pathogenesis-related 1* (*PR1*) gene [128]. *AtLBR* T-DNA single mutants (*Atlbr-1*, *Atlbr-2-1*, *Atlbr2-2*) and (*Atlbr*-DKO) double-knockouts, generated by crossing *Atlbr-1* and *Atlbr-2-1* plants, were defective in ROS generation and in upregulation of LPS-induced *PR1* expression. A transcriptome analysis revealed that AtLBR-2 plays an indispensable role in the upregulation of 65 genes associated with defence responses upon *Pseudomonas* LPS treatment [136]. Furthermore, there was a defect in the upregulation of defence-related genes and salicylic acid (SA)-mediated signalling in the *Atlbr-2-1* mutants, compared to the WT after *Pseudomonas* LPS treatment.

Lastly, a recent study has reported OsCERK1, the chitin co-receptor, as an LPS receptor/co-receptor in rice, but not in *A. thaliana* [137]. This indicates a significant difference between LPS perception in rice and Arabidopsis.

#### 5.1.4. Peptidoglycan Perception

Rice chitin elicitor-binding protein (CEBiP) homologs in Arabidopsis, LYM1 and LYM3 RLPs, bind in a ligand-specific manner to PGNs, heteropolymers that are part of the building blocks of the cell walls of Gram-negative and Gram-positive bacteria [87]. There was a significant expression of the immune marker gene, flagellin-induced receptor kinase *FRK1,* upon treatment of Arabidopsis with PGNs from Gram-negative *Pst* DC3000 [87]. Homozygous T-DNA insertional mutants of *LYM1* and *LYM3* showed a strongly reduced PGN-inducible marker gene expression and were more susceptible to *Pst* DC3000 bacterial infection than the WT (Table 1). The same authors further reported the involvement of AtCERK1 in the LYM1/LYM3 perception of PGN and subsequent immune response in *A. thaliana*. Thus, AtCERK1 is the additional protein that provides the cytoplasmic kinase domain that is lacking in the LYM1/LYM3 RLPs needed for the downstream transphosphorylation of PGN signalling. The study suggests that Arabidopsis senses PGNs in a LYM1/LYM3 and AtCERK1-dependent manner, similar to the chitin perception system in rice that uses OsCEBiP and OsCERK1 [79]. Furthermore, the direct interaction of PGN with AtLYM1 and AtLYM3 has been reported, but not with AtCERK1 [87]. In rice, OsCERK1 associates with LysM motif-containing proteins (LYP4 and LYP6) in PGN-induced defence responses [80,91,138]. Importantly, the putative ability of CERK1 to participate in the recognition and signalling of more than one MAMP supports the hypothesis of the capability of one PRR/co-receptor to recognise more than one MAMP, which favours the generation of transgenic crop plants with enhanced/altered recognition capabilities [8].

### 5.2. Fungal MAMPs

#### Chitin Perception

Chitin is a β(1→4)-linked polymer of N-acetylglucosamine, a major structural component in the exoskeleton of arthropods and cell walls of fungi [21]. Chitin and its fragments (chitooligosaccharides) are MAMPs recognised by plants PRRs, which elicit defence responses such as the oxidative burst, protein phosphorylation, transcriptional activation of defence-related genes and phytoalexin biosynthesis [21,78,139]. Kaku et al. [78] isolated and characterised a chitin elicitor binding protein (OsCEBiP), an RLP involved in the perception of chitin oligosaccharides in cultured rice cells. It was observed that OsCEBiP has two LysM motifs and a C-terminal transmembrane domain, however no intracellular kinase domain, suggesting that it requires additional protein-partner(s) to perform a signal transduction role. In rice, the LysM receptor, OsCEBiP, binds to the chitin oligosaccharide and forms a complex with OsCERK1 to trigger defence response [79]. Conversely, in *A. thaliana*, the LysM receptor, AtCERK1, directly binds to chitin, dimerises and triggers immune responses [140]. In the extracellular domain, OsCERK1 has one LysM motif, whereas AtCERK1 has three LysM motifs [15,79] that mediate the binding of chitin [141,142]. There are five genes encoding LysM receptor-like kinases (LYKs), which are comprised of *LYK1 (CERK1*) and *LYK2 - LYK5*, and three genes encoding LysM receptor-like proteins (LYPs) in the *A. thaliana* genome [143]. In this regard, LysM receptor-like kinase 1 (LysM RLK1) is involved in chitin signalling and fungal resistance in Arabidopsis [144]. A T-DNA insertion knockout mutant of *LysM RLK1* blocked the induction of chitooligosaccharide-responsive genes (CRGs) by chitooligosaccharides and increased susceptibility to fungal pathogens, but not bacterial pathogens. This was reversed when the mutant was complemented with the WT *LysM RLK1* gene using the cauliflower mosaic virus (CaMV) 35S promoter [15,144]. CERK1 KO mutants were unable to respond to chitin oligosaccharide elicitors when compared to the WT that exhibited rapid generation of ROS in Arabidopsis [15].

LYK4 is another LysM-RLK involved in chitin signalling and plant immunity in *A. thaliana*, and possibly in the chitin recognition receptor complex [89]. *LYK4* mutants were defective in the activation of chitin-responsive genes and were also susceptible to bacterial and fungal pathogen infection. Cao et al. [90] showed that AtLYK5 binds chitin with higher affinity and forms a chitin-induced complex with AtCERK1, thereby triggering immunity in *A. thaliana*. The higher affinity of AtLYK5 for chitin, compared to AtCERK1, also suggests the former to be the major chitin binding protein in Arabidopsis. These authors [90] further reported involvement of AtLYK5 in chitin-induced AtCERK1 phosphorylation and homodimerisation [90]. While *Atlyk5-2* was significantly impaired in chitin signal responses, *Atlyk4*/*Atlyk5-2* double mutant resulted in a complete loss of chitin response, indicating an overlap of signal function between AtLYK5 and AtLYK4.

The LysM domain-containing glycosylphosphatidylinositol-anchored protein 2 (LYM2), one of the three CEBiP homologs in Arabidopsis, binds chitin and mediates a reduction in molecular flux via the plasmodesmata [88]. AtCERK1 is not involved in the chitin-mediated regulation of plasmodesmata flux, thereby suggesting the presence of an alternative novel disease resistance mechanism in Arabidopsis [88,145]. Shinya et al. [146] showed that the Arabidopsis LYM2 can recognise chitin oligosaccharides in a similar way as the rice OsCEBiP, but does not participate in chitin signalling. The KO mutant of *LYM2* (*lym2-1*) was shown to be incapable of chitin-induced plasmodesmata flux, and susceptible to fungal pathogens (*Botrytis cenerea* and *Alternaria brassicicola*), but exhibited chitin-induced MAPK activation and an oxidative burst when compared to the WT [88,146]. LysM RLK1-interacting kinase 1 (LIK1) interacts with CERK1 and regulates chitin-induced MTI in Arabidopsis [147]. *LIK1* mutants (*lik1-1*, *lik1-2*, *lik1-3* and *lik1-4*), with T-DNA insertions located in intron 2, 13, and exon 18, respectively, showed an enhanced response to both chitin and flagellin elicitors. Furthermore, the mutants were defective in the expression of genes involved in jasmonic acid (JA) and ethylene (ET) signalling pathways, that have been shown to mediate resistance to necrotrophic pathogens [148]. In *A. thaliana* a powdery mildew-resistant kinase 1 (PMRK1), a RLK that is localised in the plasma membrane, is responsible for early chitin-induced defence signals against fungal pathogens [149]. *PMRK1* KO mutant (*pmrk1*) was more susceptible to both *Golovinomyces cichoracearum* and *Plectosphaerella cucumerina* (Table 1). In fact, these numerous identified PPRs linked to chitin perception suggest the complexity of plant chitin defence signalling.

## 6. BAK1 and Other Associated Proteins in MAMP Signalling

Following MAMP perception, PRRs trigger downstream events involving protein association/dissociation. BAK1 was initially identified as a co-receptor in BRI1-mediated brassinosteroid (BR) signalling, which modulates plant growth and development [150,151]. Studies have shown that BAK1 and related somatic embryogenesis receptor kinases (SERK) proteins associate with other LRR-RLKs or LRR-RLPs, and regulate plant growth and immunity [98,152,153].

Table 2 outlines different BAK1 and other associated proteins, and the implication of their KOs in plant MTI. In *A. thaliana*, both FLS2 and EFR form a complex with the co-receptor BAK1 to elicit immune responses immediately upon flg22 or elf18 perception, respectively [96,98,99,154]. Plants carrying *BAK1* mutants (*bak1-3* and *bak1-4*), generated by T-DNA insertion, displayed abnormal early and late flagellin-triggered responses [96,98]. In this regard, there was a significant reduction in the oxidative burst triggered by elf26 in *BAK1* mutants, indicating that EF-Tu is also affected by the mutation in BAK1 [96]. Interestingly, *BAK1* mutants were not completely impaired to flg22 or elf18 perception, indicating that BAK1 was not the only rate-limiting component and therefore suggests additional regulatory protein(s), such as BKK1, that are part of the FLS2 and EFR receptor complexes [96,98,152]. BAK1-disrupted *N. benthamiana* plants displayed decreased induction of MTI responses by the csp22 peptide (part of bacterial cold-shock protein) and INF1 (an oomycete elicitor) [98]. Furthermore, Arabidopsis *BAK1* KO mutants exhibited increased susceptibility to necrotrophic fungal pathogens, such as *Botrytis cinerea* and *Alternaria brassicicola* [155]. These results suggest a central role for BAK1 in modulating other PRRs besides FLS2 and EFR in plant defence signalling. The exact mechanism by which BAK1 mediates defence signalling is, however, not resolved. A recent study also showed BAK1 involvement in the tomato FLS3 recognition of flgII-28 (another flagellin epitope) and resulting immune response signalling [85].

BAK1 and BAK1-LIKE1 (BKK1) have dual physiological roles by positively regulating a BR-dependent plant growth pathway, and negatively regulating a BR-independent cell-death [156]. Here, cell death-control mediated by BAK1 and BKK1 is SA-dependent [157]. Upon flagellin perception, BIK1 as a RLCK, associates with the FLS2-BAK1 receptor complex to initiate plant innate immunity and cell death [28]. There was a significant loss of flg22-induced resistance to *Pst* DC3000 infection in *BIK1* mutant seedlings, however, the mutation did not affect flg22-induced FLS2 and BAK1 association. On the other hand, *BIK1* mutants were susceptible to necrotrophic pathogens but were resistant to a virulent bacterial pathogen *Pst* DC3000 [158]. Chen et al. [159] demonstrated that the *bik1* mutant displayed a strong SA-dependent resistance to *Plasmodiophora brassicae*, an obligate biotroph protist that induces gall formation in cruciferous plants. *Bak1-4 bik1* double mutants exhibited increased expression of plant defence genes and cell death phenotypes compared to *BIK1* single mutant [72], highlighting the cooperativity of BIK1 and BAK1 influence in plant immunity.

BIR2, a novel LRR-RLK, interacts with BAK1 in a kinase-dependent manner, and negatively regulates BAK1-dependent MAMP-triggered immune signalling [71]. Upon ligand binding to FLS2, BAK1 is released from BIR2 and recruited to the FLS2 complex. Therefore, BIR2 inhibits autoimmune cell-death responses by keeping BAK1 under control. Gao et al. [102] showed that BIR1, a BAK1-interacting RLK, negatively regulates multiple plant resistance signalling responses, and suppresses cell death in Arabidopsis. *BIR1* KO mutants (*bir1-1*) showed activation of constitutive defence responses and extensive cell death. However, the LRR-RLK SUPPRESSOR OF BIR1-1 (SOBIR1) and BAK1 function as co-receptors for LRR-RLPs, BAK1, and not SOBIR1, acts a co-receptor for LRR-RLKs [154]. SOBIR1, a co-receptor/adaptor for LRR-RLPs recruits BAK1 to SOBIR1-RLP23 and SOBIR1-RLP30 complex upon nlp20 and Sclerotinia culture filtrate elicitor1 (SCFE1) perception, respectively, in Arabidopsis [17,160]. Here, *SOBIR1* mutant (*sobir1-12*) was more susceptible to fungal *Sclerotinia sclerotiorum* and *B. cineria* [160]. The dissociation of BIR1 upon MAMP recognition by PRRs allows BAK1 to form an active complex with SOBIR1, which triggers downstream cell death and defence signalling [103].

The Arabidopsis malectin-like LRR-RLK, IMPAIRED OOMYCETE SUSCEPTIBILITY1 (IOS1) associated with PRRs FLS2, EFR and CERK1 in BAK1-dependent and -independent MTI responses [104]. Arabidopsis *IOS1* mutant (*ios1-2*) showed perturbations in the latter, including defective chitin responses and delayed upregulation of the PTI marker gene *FLG22-INDUCED RECEPTOR-LIKE KINASE1* (*FRK1*), as well as reduced downy mildew infection [103]. The malectin-like RLK FERONIA (FER), facilitates the ligand-induced complex formation of PRRs in Arabidopsis [105,161]. As such, the EFR/FLS2-BAK1 complex formation has been shown to be promoted by FER and inhibited by Rapid Alkalinization Factor 23 (RALF23) [105]. Furthermore, a *FER* mutant (*fer-4*) showed diminished ligand-induced EFR/FLS2 complex formation, with the co-receptor BAK1. In addition, AtFER is involved in the negative regulation of jasmonic acid (JA) and coronatine (COR) signalling [162]. In support, BAK1 and other defence-responsive proteins were identified in *A. thaliana* plasma membranes after *B. cepacia* and *E. coli* LPS treatments [134,135]. Here, proteins identified were similar to some previously implicated proteins upon flg22 elicitation, suggesting that LPS perception and signalling could likely resemble that of flg22.

## 7. Conclusions and Future Perspectives

MAMPs reveal the presence of pathogenic microbes, leading to the activation and upregulation of basal defence responses in plants. PRRs play a central role in MAMP recognition and the activation of downstream defence signalling. Several PRRs have been identified and classified, although the molecular mechanism(s) underlying perception of MAMPs in plants remains elusive in most cases. We have outlined several PRRs with their cognate MAMPs, and further presented the subsequent response perturbations in KO mutants, as well as those of BAK1 and other associated proteins. In addition, we reviewed the role that membrane raft domains play, in which PRRs have been suggested to be localised during the perception response, using flg22-mediated PRR-dependent ROS production in MTI as an example to set the stage for subsequent KO studies. From this review, it is clear that there is a need for more KO studies targeting the genes encoding other PRR, as well as co-receptor/associated proteins, to clearly unravel their roles in plant defence signalling. By comparing the phenotypes of wildtype (WT) and KO mutant lines, valuable information of a functional nature can be gained to support engineering of enhanced or new perception capabilities that would lead to an increase in quantitative immune responsiveness, ultimately in order to contribute towards amelioration of crop plant losses due to pathogen attack. As such, the review supports the notion that investigation of the PRR-co-receptor complex, as well as that involving other defence-associated proteins by KO studies, can enhance the understanding of MTI and the possible generation of pathogen-resistant crops.

## Abbreviation

BAK1Brassinosteroid insensitive 1 (BRI1) associated receptor kinase 1EFR Elongation factor-thermo unstable receptorETI Effector-triggered immunityFLS2 Flagellin-sensitive 2KO KnockoutLPS LipopolysaccaridesLRR Leucine-rich repeatMAMP Microbe-associated molecular patternMTI MAMP-triggered immunityPRR Pattern recognition receptorRLCK Receptor-like cytoplasmic kinaseRLK Receptor-like kinaseRLP Receptor-like proteinT-DNATransfer DNA

## Figures and Tables

**Table 1:** Defence-related PRRs and associated protein KOs in *A. thaliana*.

Receptors/Associated Proteins	Ectodomain Motif *	Family	KO Line(s)	Function	References
**Bacterial MAMPs**					
FLS2	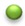	RLK	*fls2*, *fls2-101*	Recognition of flagellin (flg22 epitope)	[81,118]
EFR	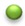	RLK	*efr-1*, *efr-2*	Recognition of EF-Tu (elf18 epitope)	[14,121]
LYM1 and LYM3	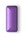	RLP	*lym1-1*, *lym1-2* and *lym3-1*, *lym3-2*	Recognition of PGNs	[87]
LBR-1	NTD	BPI/LBP	*lbr-1*	Recognition of LPS	[128]
LBR-2	NTD	BPI/LBP	*lbr-2-1*, *lbr-2-2*	Recognition of LPS	[128,136]
LORE	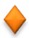	RLK	*lore*	Recognition of mc-3-OH-FAs	[93,129]
**Fungal MAMPs**					
AtLYK1 (AtCERK1)	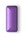	RLK	*lyk1, cerk1-1*, *cerk1-2*	Recognition of chitin	[15,79,144,146]
LYM2	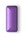	RLP	*lym2-1*	Recognition of chitin	[88,146]
LYK4	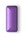	RLK	*lyk4*	Recognition of chitin	[89]
LYK5	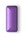	RLK	*lyk5-2*	Recognition of chitin	[90]
LIK1	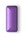	RLK	*lik1-1*, *lik1-2*, *lik1-3*, *lik1-4*	Interacts with CERK1	[147]
PMRK1	NTD	RLK	*PMRK1*	Recognition of chitin	[149]

* Coloured symbols as defined in Figure 3. NTD = N-terminal domain.

**Table 2:** Defence-related BAK1 and other associated protein KOs in *A. thaliana*.

Co-Receptor(s)/Proteins	Ectodomain Motifs *	Family	KO Line(s)	Function	References
BAK1	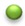	RLK	*bak1-3*, *bak1-4*	Co-receptor for PRRs	[96,98,155]
BIR1	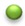	RLK	*bir1-1*	Suppresses cell death; Negative regulator of MTI	[120]
SOBIR1	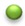	RLK	*sobir1-12*	Adaptors/co-receptor for LRR-RLPs, activates immune response	[160]
BIR2	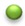	RLK	*bir2-1*	Negative regulator of MTI	[71]
BIK1	None	RLCK	*bik1*	Modulator of MTI	[72,158,159]
IOS1	Malectin-like	RLK	*ios1-2*	Scaffold for PRR	[103]
BKK1	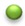	RLK	*bkk1*	Regulator of cell death	[156]
FER	Malectin-like	RLK	*fer-4*	Scaffold for PRR	[105]

* Coloured symbols represent Leucine-rich repeats.
